# Psychological outcomes of depression after legally enforced quarantine during the COVID-19 pandemic: a cross-sectional study

**DOI:** 10.1186/s12889-025-25751-0

**Published:** 2025-12-03

**Authors:** Alisa Fabrice, Lisa Klee, Sven Feddern, Chiara Gabriel, Anna Carlotta Graf, Annelene Kossow, Johannes Niessen, Nikola Schmidt, Gerhard A. Wiesmüller, Barbara Grüne, Christine Joisten

**Affiliations:** 1 Cologne Health Department, Infektions- und Umwelthygiene, Neumarkt 15–21, Trajanstr. 43, Köln, 50667 Germany; 2 Institute for Occupational Medicine and Social Medicine, University HospitalMedical FacultyRWTH Aachen University, Aachen, Germany; 3 Institute of Hygiene, University Hospital Muenster, Albert-Schweitzer-Campus 1, Münster, 48149 Germany; 4 Department for Physical Activity in Public Health, Institute of Movement and Neurosciences, German Sport University Cologne, Am Sportpark Müngersdorf 6, Cologne, 50933 Germany

**Keywords:** Public health department, COVID-19 measures, Depression, Legally enforced quarantine, Psychological outcomes, Quarantine

## Abstract

**Background:**

Legally enforced quarantines during the COVID-19 pandemic resulted in increased psychological distress, particularly among individuals with pre-existing mental health conditions. In this study, part of the Cologne Corona Counselling and Support for Infected and Contact Persons During the Quarantine Period (CoCo-Fakt) project, we hypothesised that individuals with pre-existing depression experienced greater psychological distress during legally imposed quarantine/isolation and thus require additional support. We also aimed to explore whether coping mechanisms and resilience can enhance the care provided to at-risk individuals facing similar situations.

**Methods:**

This study included 10,033 participants (infected and contact persons) registered with the Cologne Public Health Department between December 2020 and January 2021. We collected data via online questionnaires covering demographics, mental health status, coping strategies, resilience, and support system usage.

**Results:**

Individuals suffering from depression (ISFDs, *n* = 260) reported significantly higher psychological distress scores than individuals not suffering from depression (INSFDs, *n* = 9773; *p <* 0.001). ISFDs also reported significantly lower coping (*p <* 0.001) and resilience (*p <* 0.001) scores and used support systems approximately six times more often. Backwards elimination regression analysis revealed significantly higher psychological distress scores among individuals who were female, were younger, had higher educational level, had COVID-19, and had a chronic disease other than depression. No significant interactions were found in the moderation analyses. The model accounted for 31.2% of the observed variance.

**Conclusions:**

The findings suggest the need for tailored interventions to help both ISFDs and INSFDs develop coping strategies and resilience during quarantines. Ensuring that sufficient therapeutic services and support are available for ISFDs is crucial for maintaining good mental health.

**Supplementary Information:**

The online version contains supplementary material available at 10.1186/s12889-025-25751-0.

## Introduction

 First detected in Wuhan, China, in December 2019, severe acute respiratory syndrome coronavirus type 2 (SARS-CoV-2) is a highly infectious beta coronavirus and the cause of the coronavirus disease 2019 (COVID-19) pandemic [1]. Before the availability of vaccines and the subsequent implementation of active immunisation against SARS-CoV-2, the primary strategy for mitigating the spread of the virus was to immediately isolate infected persons (IPs) and their contact persons (CPs) [2, 3]. While these isolation measures were essential for ensuring public health, they also profoundly impacted affected individuals’ psychological well-being, leading to issues such as feelings of loneliness [4–6], depression [7–9], anger [4, 10], stress [9–12], anxiety [8–10, 13], stigmatisation [5, 11, 13] and post-traumatic stress disorder [5, 7].

Initiated in March 2020, the German COVID-19 Snapshot Monitoring (COSMO) study regularly monitored a representative sample of the German population to assess the COVID-19 pandemic’s psychological impact, particularly in terms of stress, anxiety and depression levels [14]. Its findings suggested that factors such as young age, female sex, parenthood and having a migrant background may be potential risk factors for psychological morbidity [15]. Similarly, an Australian online survey conducted in July 2020 involving 587 participants reported an increase in psychological distress related to the COVID-19 pandemic, particularly among those who were middle-aged (aged 30–59 years), had pre-existing mental disorders, were female, and had a greater fear of the virus [16]. In their systematic review, Yuan et al. [17] found that mental disorders, female sex and alcohol consumption were associated with a higher prevalence of depression and anxiety in the general population.

Most studies that have assessed the psychological outcomes of the COVID-19 pandemic have focused on the consequences of general lockdown measures; however, there is a paucity of research on the specific effects of legally mandated quarantines or isolation on IPs and CPs. Therefore, in this study, we aimed to address this gap, hypothesising that individuals with pre-existing depression experienced greater psychological distress during legally imposed quarantine (CPs) or isolation (IPs) and thus require additional support. We also aimed to explore whether coping mechanisms and resilience can enhance the care provided to at-risk individuals facing similar situations. By investigating these factors, we aim to develop actionable recommendations for future interventions.

## Methods

### Study design

We conducted the first wave of the Cologne Corona Counselling and Support for Infected and Contact Persons During the Quarantine Period (CoCo-Fakt) study between 12 December 2020 and 6 January 2021 [18]. The CoCo-Fakt online monitoring study involved individuals who had tested positive for SARS-CoV-2 and their confirmed contacts who were registered in the Digital Contact Management (DiKoMa) database, which was developed by the Office for Information Processing of the city of Cologne, Germany [19]. The DiKoMa database also included individuals who returned to Cologne from areas at high risk of COVID-19 or who had an activated SARS-CoV-2 warning smartphone application. Developed by the Robert Koch Institute and introduced in Germany in June 2020 [20], this application was used for contact tracing in the context of COVID-19. The application was activated when it was determined that the individual had likely been in close contact with an IP, and transmission could not be ruled out. While these individuals were also registered with the public health department, they were only classified as an IP if SARS-CoV-2 detection was conducted immediately or as a CP if they had had verifiable contact with an IP. Otherwise, their data remained in the DiKoMa database but were not integrated into the CoCo-Fakt analyses.

The other exclusion criteria were:

- failure to provide consent to participate.
- being younger than 16 years or providing implausible age information (e.g. if their age was given as ‘214’).
- not providing information about the rationale for their quarantine status.
- being pregnant (they received an expanded questionnaire tailored to their condition).
- being unable to complete the questionnaire independently, and.
- providing no information about their depression status.

Therefore, this sub-analysis included individuals who:

- had given their consent to participate.
- were aged 16 years or older.
- stated their sex.
- were placed in quarantine as an IP or CP.
- could complete the questionnaire independently, and.
- provided information about their depression status.

The survey was conducted in accordance with the latest version of the Declaration of Helsinki [21] using Unipark software (Tivian XI GmbH, Cologne, Germany). It took approximately 30 min to complete. Ethical approval for the CoCo-Fakt study was obtained from the Rhenish-Westphalian Technical University Aachen (approval number: 351/20).

### Participants

The email addresses of 36,498 individuals were extracted from the DiKoMa database. Of these individuals, the survey link was sent to 33,699, of whom 13,155 clicked on the survey link. Following the application of the exclusion criteria, 3,122 questionnaires were deemed unfit for consideration: 98 questionnaires were excluded because the respondents were pregnant and thus received a tailored questionnaire. Another 1,586 questionnaires were excluded because no age was specified (*n* = 1,572), the age given was implausible (*n* = 9), or the respondents were younger than 16 years (*n* = 5). In addition, 125 questionnaires were excluded due to missing information on the sex (*n* = 89) or the answer was ‘other/prefer not to say’ (*n =* 36). Moreover, 962 questionnaires were excluded because the respondents were neither IPs nor CPs, including those who had to quarantine since they had returned from a high-risk area (*n* = 59), those who were in quarantine due to an activated SARS-CoV-2 warning smartphone application (*n* = 29), those who were quarantined for another reason or did not know why they were quarantined (*n* = 106), and those who did not provide any information about their reasons for quarantine (*n* = 753), as well as questionnaires not completed by the IP or CP themselves but by their legal representatives (*n* = 15). Finally, 351 questionnaires were excluded because the respondents did not provide any information about their depression status.

**Fig. 1:**
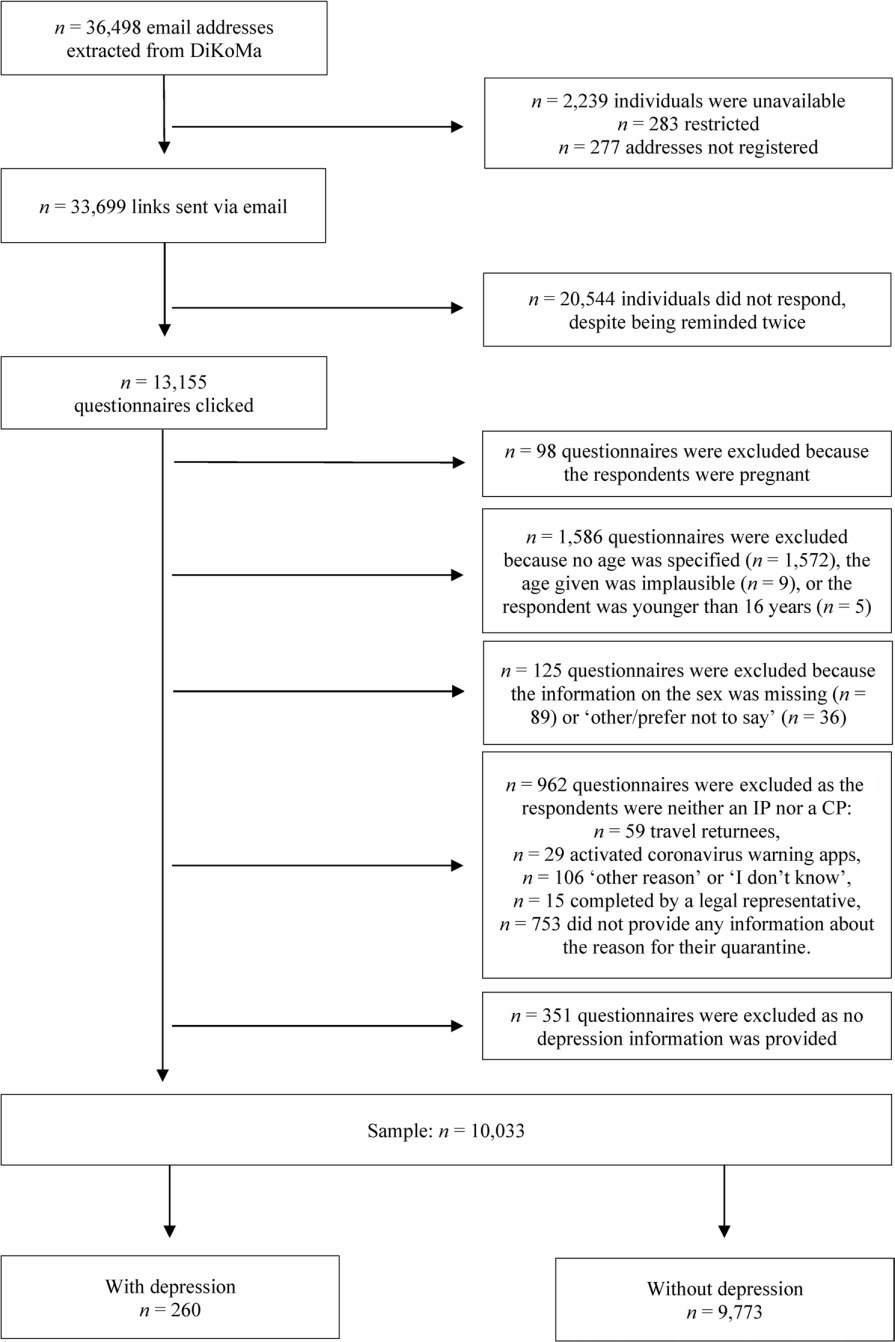
Study sample

Therefore, the subgroup analysis included 10,033 participants, of whom 260 (2.6%) reported having depression; however, due to the complexity of the CoCo-Fakt survey, we did not distinguish between specific forms of depression, such as major depression, seasonal affective disorder or bipolar disorder. In total, 3,860 participants (38.5%) were IPs and 6,173 (61.5%) were CPs. In addition, 5,999 participants (59.8%) were female. The participants’ ages ranged from 16 to 93 years, with a mean of 40.7 years (standard deviation [SD] = 14.2 years, Fig. 1).

The period between the commencement of quarantine and participation in the survey ranged from 1 to 330 days (mean = 98.2 days, SD = 85.8). The questionnaire primarily referred to participants’ circumstances at the time of its completion. If questions referred to a previous period, the participants were explicitly instructed to provide answers reflecting their conditions during that specific time frame.

### Questionnaire

The questionnaire assessed quantitative and qualitative variables. It was designed based on the COSMO study from the University of Erfurt, Germany [14, 22], thereby allowing for the comparison of data from the COSMO study on the general population’s experience of COVID-19 pandemic-related restrictions with data from individuals in legally mandated quarantine or isolation (IPs and CPs). The questionnaire was made available in German, Turkish, and English. The responses to the individual questions were entirely voluntary; no question required an answer for the questionnaire to be submitted. The following factors were recorded (see Additional File 1).

#### Demographics

The participants were asked for their age, sex and educational level. Their educational level was categorised as high, middle, or low according to the socio-economic status by the study of adult health in Germany (DEGS1) [23]. The questionnaire also asked about their migration background and health status. For health status, the participants were asked if they had any chronic diseases; if they answered ‘yes’, they were asked to specify the type of illness. Based on their self-reported answer regarding depression, the participants were categorised as either suffering from depression (ISFDs, individuals suffering from depression) or not suffering from depression (INSFDs, individuals not suffering from depression). Since no formal screening tool or clinical verification was used to assess depression, the classification relied solely on the participants’ subjective self-assessment.

#### Personal circumstances

Items asking about the participants’ personal circumstances were derived from the German Health Interview Survey for Adults [24]. For example, these items asked whether the participants were living with a partner, had children, and had a garden or balcony.

#### Reason for isolation or quarantine

The participants were asked about the reason for their quarantine and based on their answers, categorised as either IPs (‘I tested positive for the coronavirus’ or ‘I was a contact person and tested positive for the coronavirus afterwards’) or CPs (‘I was a contact person’ or ‘I was a contact person several times’). All other possible answers were excluded from this analysis (see Additional File 1).

#### Psychological distress during quarantine or isolation

The participants’ psychological situation experienced during quarantine was assessed using a series of statements adapted from wave 4 of the COSMO survey (24–25 March 2020), subsection 7 [25]. The participants were instructed to refer specifically to their psychological experiences during the quarantine period when responding to these statements. These statements were as follows:

1. ‘I felt nervous, anxious, or tense.’
2. ‘I felt down or depressed.’
3. ‘I felt lonely.’
4. ‘I thought of the future with hope.’
5. ‘Thoughts of my experiences during the coronavirus pandemic triggered physical reactions in me, such as sweating, shortness of breath, dizziness or palpitations.’

The items were answered on a 6-point Likert scale from 1 (*not at all or less than one day*) to 6 (*always or daily*). Notably, the answers to Item 4 were reverse-coded. Then, the psychological distress score was calculated by taking the mean of all items (possible range: 1–6), with a lower score indicating lower psychological distress. Cronbach’s alpha for the psychological distress score was 0.694 [26].

#### Coping

The coping score was based on wave 5 of the COSMO survey (31 March–1 April 2020), subsection 29 [27], and comprised the following six items. The participants were instructed to consider how they coped specifically during the quarantine period when responding to these statements:

1. ‘I received offers of support from family, friends or neighbours.’
2. ‘I had a plan for my daily life in terms of sleep, work and physical activity.’
3. ‘I discovered activities for myself that made staying home easier.’
4. ‘I used digital media to communicate with family, friends and acquaintances.’
5. ‘I was bored.’
6. ‘There was nothing I could do myself to positively influence the situation.’

The items were again answered on a 6-point Likert scale from 1 (*does not apply at all*) to 6 (*fully applies*). The answers to Items 5 and 6 were reverse-coded. Then, the coping score was calculated by taking the mean of all six items (possible range: 1–6), with a higher score indicating greater coping competence. Cronbach’s alpha for the coping score was 0.685 [26].

#### Resilience

The Brief Resilience Scale (BRS) was designed to assess resilience and comprises six bipolar items (three negatively- and three positively-structured questions) [28]. The participants were asked to reflect on their general resilience abilities without specific reference to the quarantine period:

1. ‘I tend to bounce back quickly after difficult times.’
2. ‘I have a hard time getting through stressful situations.’
3. ‘I don’t need a lot of time to recover from a stressful event.’
4. ‘It’s hard for me to go back to normal when something bad has happened.’
5. ‘I usually get through difficult times without any major problems.’
6. ‘It tends to take me a long time to get over setbacks in my life.’

The items were originally answered on a 5-point Likert scale; however, we changed them to a 6-point Likert scale ranging from 1 *(I totally agree*) to 6 (*I totally disagree*) to align this section of the questionnaire with the other sections. The answers to Items 1, 3 and 5 were reverse-coded. The score was calculated according to Smith et al.’s suggestion [28]. A higher score indicated higher resilience. Cronbach’s alpha was 0.812 in this study [26].

#### Support systems

The participants were asked two questions about their support systems, with instructions to specifically refer to the quarantine period:

1. ‘Did you make use of professional support systems, such as helplines?’
2. ‘If so, which professional support systems did you use?’

While *yes/no* answers were permitted for the first question, up to three free-text fields were allowed for the second question. To evaluate the responses, 13 categories were defined, with two independent reviewers assigning each free-text response to one of these categories, and all 13 categories were used (see Additional File 2).

The participants were also invited to state their wishes for further measures: ‘What further measures would you like to see or would you have liked to have seen after the quarantine period?’ They could again answer this question in a maximum of three free-text fields. In the analysis, their free-text responses were evaluated according to 20 categories, per Maertl et al. [29], with two independent reviewers assigning them to the appropriate categories (see Additional File 3).

### Data analysis

The data were analysed using SPSS Statistics (version 29.0), and a *p* < 0.05 was considered statistically significant. Continuous variables are reported as means (SDs), and categorical variables are reported as numbers (percentages). The variables were compared between groups using Pearson’s chi-squared test and the independent samples *t*-test. Effect sizes were estimated using Cohen’s *d* for independent *t*-tests (trivial: <0.2, small: 0.2–<0.5, moderate: 0.5–<0.8, and large: ≥0.8) or Cramer’s *V* for chi-squared tests (small: <0.3, moderate: 0.3–<0.5, and large: ≥0.5) to identify significant differences. Notably, while not all participants answered all questions, all available answers were included in the descriptive statistical analysis. Only the scores of those participants who fully answered all sub-questions for the respective section were included in the analysis (psychological distress score: *n* = 7,960; coping score: *n* = 7,958; BRS: *n* = 8,246). Stepwise backwards elimination multiple regression analysis was performed to assess the factors influencing psychological distress. First, whether the model met the assumptions for linear regression was checked. The independence of residuals was assessed using the Durbin–Watson test; the value of the Durbin–Watson test was 1.999, indicating that the model had no autocorrelation. Multicollinearity was assessed using the variance inflation factor (VIF), the VIF values of all predictors in the final model were < 10, indicating no multicollinearity. To assess a possible interaction effect, moderation analyses were performed with depression (*yes/no*) as the moderator variable and two predictors: coping score and BRS score. The model included additional independent variables – the reason for quarantine, age (in years) sex, migration background, educational level, the presence of a chronic disease other than depression and living with a partner – which were tested for the independence of residuals and multicollinearity. Only participants who provided all this information were included in the regression (*n* = 7,677).

## Results

### Demographics, personal circumstances and reasons for isolation or quarantine

Depression status did not differ significantly between IPs and CPs (*χ*^2^_(1)_ = 0.810, *p =* 0.368); therefore, these subgroups were examined together in this study. ISFDs and INSFDs differed significantly in sex, age, educational level, migration background, the presence of a chronic disease other than depression, and living with a partner, all effect sizes were small (*d* < 0.5 and *V* < 0.3, Table 1).

**Table 1:** Demographics, personal circumstances, and reasons for isolation or quarantine

	Total	ISFD	INSFD	Statistic	df	*p*-value	Effect Size
Sample size (*n* [%])	10,033 (100)	260 (2.6)	9,773 (97.4)				
Sex (*n* [%])				5.645^a^	1	0.018	0.024^d^
Female	5,999 (59.8)	174 (66.9)	5,825 (59.6)				
Male	4,034 (40.2)	86 (33.1)	3,948 (40.4)				
Age (years)				−5.228^b^	10,031	< 0.001	0.329^c^
Mean (SD)	40.7 (14.2)	45.2 (13.8)	40.6 (14.2)				
Range	16–93	18–83	16–93				
Educational level (*n* [%])				35.764^a^	2	< 0.001	0.060^d^
High	7,994 (80.3)	173 (67.3)	7,821 (80.6)				
Medium	1,872 (18.8)	76 (29.6)	1,796 (18.5)				
Low	91 (0.9)	8 (3.1)	83 (0.9)				
Chronic disease other than depression (*n* [%])				644.662^a^	1	< 0.001	0.253^d^
Yes	2,122 (21.2)	220 (84.6)	1,902 (19.5)				
No	7,911 (78.8)	40 (15.4)	7,871 (80.5)				
Migration background (*n* [%])				3.218^a^	1	0.073 n.s.	
Yes	527 (5.3)	20 (7.8)	507 (5.3)				
No	9,326 (94.7)	235 (92.2)	9,091 (94.7)				
Living with a partner (*n* [%])				6.724^a^	1	0.010	0.026^d^
Yes	7,000 (71.3)	164 (64.1)	6,836 (71.5)				
No	2,818 (28.7)	92 (35.9)	2,726 (28.5)				
Children (*n* [%])				0.034^a^	1	0.854 n.s.	
Yes	4,262 (42.7)	112 (43.2)	4,150 (42.7)				
No	5,723 (57.3)	147 (56.8)	5,576 (57.3)				
Housing situation (*n* [%])				6.026^a^	3	0.110 n.s.	
Garden	2,206 (22.1)	44 (17.0)	2,162 (22.2)*				
Balcony	5,056 (50.6)	149 (57.5)	4,907 (50.4)*				
Garden and balcony	1,200 (12.0)	28 (10.8)	1,172 (12.0)*				
Neither	1,528 (15.3)	38 (14.7)	1,490 (15.3)*				
Reason for quarantine (*n* [%])				0.810^a^	1	0.368 n.s.	
IP	3,860 (38.5)	107 (41.2)	3,753 (38.4)				
CP	6,173 (61.5)	153 (58.8)	6,020 (61.6)				

^a^Chi-squared test

^b^Independent samples *t*-test

^c^Cohen’s *d*

^d^Cramer’s *V*

*n.s.* not significant

*Rounding error

### Psychological distress, coping and resilience

The ISFDs had significantly higher psychological distress scores, lower coping scores, and lower BRS scores than the INSFDs (Table 2).

**Table 2:** Sum scores for psychological distress, coping and resilience

Sum Score	Total Sample (*n*)	Depression: Yes		Depression: No		t	df	*p*-value^a^	d
		*n*	mean (SD)	*n*	mean (SD)				
Psychological distress	7,960	216	1.6 (0.8)	7,744	1.0 (0.7)	−13.397	7,958	< 0.001	−0.924
Coping	7,958	215	4.1 (1.3)	7,743	4.6 (1.0)	6.050	221,084	< 0.001	0.536
BRS	8,246	223	3.0 (1.0)	8,023	4.4 (1.0)	20.101	8,244	< 0.001	1.365

*SD* Standard deviation

^a^Independent samples *t*-test

### Backwards elimination regression analysis

The final model of the backwards elimination regression analysis revealed significantly higher psychological distress scores among the ISFDs than among the INSFDs, as well as among those with lower coping and BRS scores. Psychological distress scores were significantly higher among participants who were female, younger, had higher educational level, did not live with a partner, did not have a balcony or garden, were infected with COVID-19, and had a chronic disease other than depression (Table 3). No significant interactions were found in the moderation analyses. The model accounted for 31.2% of the observed variance.

**Table 3:** The baseline and final models of the stepwise backwards elimination regression analysis of the effects on psychological distress, including moderation analyses with ISFDs versus INSFDs as the moderator variable

Model	Unstandardised Coefficients		Standardised Coefficients	t	*p*-value	95% CI		Collinearity Statistics		*R*^2^ corr.
	ß	Std. Error	ß			LL	UL	Tolerance	VIF	
Baseline model										
ISFDs vs. INSFDs^a^	0.177	0.165	0.041	1.074	0.283	−0.146	0.501	0.064	15.624	31.2%
Coping score	−0,188	0.008	−0.260	−24.379	< 0.001	−0.203	−0.173	0.802	1.246	
ISFDs vs. INSFDs * coping score	0.007	0.036	0.007	0.209	0.835	−0.063	0.078	0.074	13.465	
BRS score	−0.239	0.007	−0.345	−32.302	< 0.001	−0.253	−0.224	0.800	1.250	
ISFDs vs. INSFDs * BRS score	−0.010	0.044	−0.007	−0.216	0.829	−0.096	0.077	0.089	11.218	
Sex^b^	−0.131	0.014	−0.090	−9.084	< 0.001	−0.159	−0.102	0.939	1.065	
Age (years)	−0.002	0.001	−0.046	−3.695	< 0.001	−0.004	−0.001	0.581	1.720	
Educational level^c^	0.045	0.017	0.026	2.684	0.007	0.012	0.078	0.957	1.045	
Migration background^d^	0.030	0.032	0.009	0.956	0.339	−0.032	0.092	0.980	1.020	
Living with a partner^e^	−0.148	0.016	−0.094	−9.352	< 0.001	−0.179	−0.117	0.894	1.119	
Children^f^	0.017	0.018	0.012	0.941	0.347	−0.018	0.051	0.598	1.673	
Housing situation^g^	−0.018	0.008	−0.023	−2.175	0.030	−0.035	−0.002	0.844	1.184	
Reason for quarantine^h^	−0.095	0.014	−0.066	−6.826	< 0.001	−0.123	−0.068	0.990	1.010	
Chronic disease other than depression^i^	0.046	0.018	0.027	2.593	0.010	0.011	0.080	0.865	1.156	
Final model										
ISFDs vs. INSFDs^a^	0.179	0.044	0.041	4.032	< 0.001	0.092	0.265	0.890	1.124	31.2%
Coping score	−0.188	0.008	−0.260	−24.929	< 0.001	−0.203	−0.173	0.839	1.192	
BRS score	−0.239	0.007	−0.345	−32.820	< 0.001	−0.254	−0.225	0.823	1.215	
Sex^b^	−0.131	0.014	−0.090	−9.141	< 0.001	−0.159	−0.103	0.946	1.057	
Age (years)	−0.002	0.001	−0.041	−3.733	< 0.001	−0.003	−0.001	0.758	1.320	
Educational level^c^	0.043	0.017	0.025	2.587	0.010	0.011	0.076	0.963	1.038	
Living with a partner^e^	−0.145	0.015	−0.092	−9.355	< 0.001	−0.175	−0.115	0.934	1.070	
Housing situation^g^	−0.017	0.008	−0.021	−2.058	0.040	−0.033	−0.001	0.895	1.118	
Reason for quarantine^h^	−0.096	0.014	−0.066	−6.885	< 0.001	−0.123	−0.069	0.993	1.007	
Chronic disease other than depression^i^	0.045	0.018	0.026	2.549	0.011	0.010	0.079	0.866	1.155	

The psychological distress score was the dependent variable; *n* = 7,677; Durbin–Watson statistic = 1.999

*Std. Error *Standard error, *CI* Confidence interval, *LL* Lower limit, *UL* Upper limit, *VIF* Variance inflation factor

^a^0 = no, 1 = yes

^b^0 = female, 1 = male

^c^0 = low, 1 = middle, 2 = high

^d^0 = no, 1 = yes

^e^0 = no, 1 = yes

^f^0 = no, 1 = yes

^g^0 = neither, 1 = balcony, 2 = garden, 3 = both

^h^0 = infected person, 1 = contact person

^i^0 = no, 1 = yes

*denotes an interaction term between the respective variables in theregression model and does not indicate statistical significance

### Support systems

The ISFDs reported using support systems significantly more often than the INSFDs (12.1% vs. 2.6%; *χ*^2^_(1)_ = 68.857, *p* < 0.001, *V* = 0.093; *n*_total_ = 7,906). The free-response questions were completed by 207 participants, comprising 25 ISFDs and 182 INSFDs. Of the available support systems, mental health support systems were used the most frequently (therapist: *n =* 62; semi-professional telephonic mental health counselling: *n =* 14; psychological support by untrained staff: *n =* 9), with 42.9% (*n =* 12) of the ISFDs and 25.8% (*n =* 50) of the INSFDs reporting that they consulted a therapist for support. The second most frequently used support system was quarantine-specific support (*n* = 59, 26.6%), reported by 17.9% (*n =* 5) of the ISFDs and 27.8% (*n =* 54) of the INSFDs (see Additional File 4).

Information about participants’ wishes for further measures was provided by 134 ISFDs and 3,750 INSFDs (*n*_total_ = 3,884). The most frequently cited desire was for support from the public health department (*n* = 1,692, 36.7%), which accounted for 29.5% (*n* = 51) of the responses from the ISFDs and 37.0% (*n* = 1,641) of the responses from the INSFDs. The second most common desire was for testing (*n* = 854, 18.5%); this was stated by 13.9% (*n* = 24) of the ISFDs and 18.7% (*n* = 830) of the INSFDs. In addition, 10.2% (*n* = 471) of the responses were assigned to the category ‘medical care’, comprising 12.7% (*n* = 22) of the responses from the ISFDs and 10.1% (*n* = 449) of the responses from the INSFDs.

The largest difference between ISFDs and INFSDs was observed in the psychological support category, with 9.8% (*n* = 17) of the ISFDs mentioning a desire for access to psychological support compared to 2.2% (*n* = 98) of the INSFDs (see Additional File 5).

## Discussion

To our knowledge, this study is the first to investigate the psychological impact of legally enforced COVID-19 quarantine on adults (both IPs and CPs) with depression. Our findings confirm our hypothesis that individuals with depression (ISFDs) would experience greater psychological distress than individuals without depression (INSFDs). Additionally, coping and BRS scores were found to be lower among ISFDs. However, further analyses revealed one significant main effect, indicating that the presence of depression and lower coping or resilience scores – but not their interaction – are associated with heightened psychological distress during isolation. Additional factors contributing to psychological distress during quarantine included a positive COVID-19 test result, female sex, younger age, not living with a partner, having a higher educational level, the lack of a balcony or garden, and having a chronic disease other than depression. Our findings are consistent with those of previous studies, which have repeatedly identified female sex [6, 9, 16, 17, 30], younger age [6, 9, 16], chronic disease [9, 17], pre-existing psychological illnesses such as depression [6, 9, 16, 17, 30] and testing positive for or having symptoms of COVID-19 [17, 30] as factors associated with worse mental health outcomes.

Our findings also parallel those of the German COSMO study: In both study cohorts, social support and digital communication were vital coping strategies that were used more often by older individuals and INSFDs [31]. Conversely, younger individuals in both studies received fewer offers of support and exhibited lower resilience and a higher risk of psychological distress [32]. These parallels highlight the need for targeted interventions to enhance social support and digital communication, particularly among younger individuals with depression who may be more susceptible to psychological distress.

Globally, the prevalence of depression has increased by 28% since the start of the COVID-19 pandemic in early 2020 [33]. Effective interventions are thus crucial, particularly for vulnerable groups. Budimir et al. [34] identified positive thinking, active stress coping and social support as significant positive predictors of psychological quality of life, emphasising their protective effects against stress, depression, insomnia and anxiety disorders. Similarly, in their systematic review, Yuan et al. [17] reported that strong social support and high exercise frequency were associated with a lower risk of developing depression and anxiety symptoms. Given the established association between loneliness during the COVID-19 pandemic and an increased risk of depression [30, 35], alternative contact methods, such as social networks and digital communication are recommended to prevent social isolation [36].

Our findings revealed that individuals with depression used support systems approximately six times as frequently as those without depression. While ISFDs were mainly in contact with therapists and psychological counselling services, INSFDs preferred to use Cologne’s hotline, which provided information about quarantine regulations, symptom documentation and medical referrals. This difference might seem counterintuitive since individuals experiencing depression generally seek help less often than individuals not experiencing depression [37, 38]. One potential explanation for this phenomenon is the unique context of COVID-19 and its enforced quarantines. The increased levels of psychological distress and social isolation experienced by individuals with depression may have driven them to seek specific help. The immediate and severe impact of quarantine on their mental health could also have created an urgent need for support, making them more likely to use available resources. This hypothesis is substantiated by El Hayek et al. [39], who examined the use of telepsychiatry in Arab countries: the Jordanian Psychiatrists Association established a telephone hotline for psychological support, which was met with considerable interest and handled over 270 cases per month, highlighting the potential for such initiatives to address significant needs. It is also conceivable that support services were more visible and accessible during the COVID-19 pandemic, with more proactive outreach efforts and public health campaigns emphasising the importance of mental health support. Another potential explanation for the observed increase in contact between the ISFDs in our study and their therapists is that therapists initiated the contact, perhaps recognising their patients’ more challenging circumstances during the quarantine and contacting them more frequently. Unfortunately, the items in the questionnaire used in our study did not ask who initiated the contact.

Additionally, it has been hypothesised that the absence of psychological support or counselling during quarantine exacerbated the development of depression [40]. This effect can be inferred from the participants’ wishes for further measures: nearly 10% of individuals with depression indicated a desire for psychological support in similar situations, compared to only 2% of individuals without depression. Targeted and timely interventions may prevent the aggravation of symptoms in those with depression and mitigate psychological distress within the healthy general population [41, 42]. Consequently, in future scenarios where quarantine or isolation measures are deemed necessary, health authorities should include structured digital support measures, such as teleconsultations, mental health apps and proactive messaging systems, in their pandemic preparedness plans. These scalable, low-threshold tools could be quickly implemented in future public health crises and adapted to meet the needs of vulnerable groups, particularly those with pre-existing mental health conditions. Integrating these services into the existing infrastructure of local health authorities could enhance accessibility and continuity of care, thereby mitigating the long-term mental health consequences of legally mandated isolation.

### Strengths and limitations

One strength of our study was its large sample size: 10,033 individuals who had experienced legally mandated quarantine or isolation because they were IPs or CPs. In addition, the inclusion of free-response questions generated in-depth insights into the participants’ desires for additional measures and their use of support services.

However, our study had some limitations. Firstly, the proportion of individuals with depression in our cohort was lower than in the general German population, with nearly 3% of the participants reporting depression compared to approximately 8% of the general population in 2021 [43]. Secondly, information about depression was self-reported by participants, making verification impossible. Previous studies have indicated that self-reporting is reliable for well-defined chronic conditions, such as hypertension [44]. However, no comparable data are yet available for depression. Therefore, the use of self-reports may have distorted our results. Thirdly, our analyses did not differentiate between specific forms of depression.

Fourthly, the proportion of participants in our cohort with high education levels (80.3%) was higher than the average in the general German population (20.1%) [23]. Many studies have identified a high education level as a protective factor for mental health [45–48], whereas a low education level has been identified as a risk factor for low resilience [49]. Both mental health and resilience tended to be worse in the general population than in our study cohort.

Fifthly, the proportion of individuals with a migration background was lower in our study than in the general German population (5.3% vs. 24.3% [50]), despite the questionnaire being available in English, German, and Turkish.

Sixthly, some participants did not complete the questionnaire until weeks or months after their quarantine, so their perceptions of their experience might have changed, potentially leading to recall bias. Individuals diagnosed with depression have been observed to retrospectively perceive past stressful experiences as more distressing than they actually were during the event [51, 52]. It has been hypothesised that this phenomenon contributes to an overestimation of the severity of depression symptoms among individuals who have experienced them. The accuracy of retrospective reports is also known to decrease as the time between the event and the administration of the survey increases [53, 54] because individuals tend to recall their experiences during the quarantine period less accurately over time, which could have influenced our results. However, allowing a longer period between quarantine and completing the questionnaire led to an increase in the size of the study cohort, which could have led to more reliable results [55].

Finally, variations in the duration and circumstances of the participants’ quarantines were not recorded. For example, the duration of quarantine can vary by up to 13 days, depending on the time of infection and contact with the public health department. Additionally, variations in individual quarantine conditions, such as the size of the participants’ living space and their current level of knowledge about SARS-CoV-2, were not considered in the analyses.

## Conclusion

Despite its limitations, our study yielded significant findings that contribute to the current understanding of the psychological outcomes of depression during quarantine periods. Compared to their non-depressed counterparts, individuals with depression perceived the legally enforced quarantine measures during the COVID-19 pandemic to be a greater burden, resulting in greater psychosocial stress. They also used coping strategies less frequently, had lower resilience and relied more often on therapeutic support systems. Moderation analyses that incorporated coping responses and resilience as independent variables and depression as the moderator revealed a significant main effect but no interaction effects. Consequently, poor coping responses and low resilience were identified as predictors of increased psychological distress during quarantine and isolation, independent of depression.

To address the challenges posed by quarantine, it is essential to provide support that enhances resilience and promotes effective coping strategies for maintaining or improving mental health. Such support must be tailored to meet the needs of each recipient. Finally, it is imperative to ensure that individuals with depression have sufficient and guaranteed access to therapeutic services.

## Supplementary Information

Supplementary Material 1.

Supplementary Material 2.

Supplementary Material 3.

Supplementary Material 4.

Supplementary Material 5.

## Data Availability

The data sets used and/or analyzed in the present study are not publicly available due to the inclusion of sensitive personal data but are available from the corresponding author on reasonable request.

## Abbreviations

BRS: Brief resilience scale
CoCo-Fakt: Corona Counseling and Support for Infected and Contact Persons During the Quarantine Period
COSMO: COVID-19 Snapshot Monitoring
COVID-19: Coronavirus disease 2019
CPs: Contact persons
DiKoMa: Digital Contact Management
INSFD: Individuals not suffering from depression
IPs: Infected persons
ISFDs: Individuals suffering from depression
RWTH: Rhenish-Westphalian Technical University
SARS-CoV-2: Severe acute respiratory syndrome coronavirus type 2
