# Supplementary material for: Psychological outcomes of depression after legally enforced quarantine during the COVID-19 pandemic: a cross-sectional study
Source: BMC Public Health. 2025 Dec 3;26:38. doi: 10.1186/s12889-025-25751-0 (PMC12766936; doi:10.1186/s12889-025-25751-0)
Supplement: Supplementary file 1 — Supplementary Material 1. [file 12889_2025_25751_MOESM1_ESM.docx]

*Additional file 1: Questionnaire Items*

| **Indicators** | **Number of items (total)** | **Item format** | **Item(s)/ Source** |
| --- | --- | --- | --- |
| **Demographic data** | 7 |  |  |
| **Age** | 1 | 1 OQ |  |
| **Sex** | 1 | 1 MCQ | male/female |
| **SES** | 2 | 1 MCQ, 1 OQ | highest vocational qualification, number of school years [22] |
| **Migration background** | 1 | 1 OQ | main language spoken in the household: German = no, any other language = yes [23] |
| **Chronic Disease** | 2 | 1 Yes/No, 1 MCQ | Asthma, including allergic asthma; chronic bronchitis, chronic obstructive pulmonary disease, pulmonary emphysema; heart attack, coronary heart disease; congestive heart failure; stroke; diseases of the musculoskeletal system; diabetes – type 1 or 2, hypertension/high blood pressure; hypercholesterolemia; allergies; chronic liver diseases; chronic kidney problems or kidney failure; depression; cancer - which type; inflammatory bowel disease [23] |
| **Personal Situation** | 3 |  |  |
| **Living with a partner** | 1 | 1 Yes/No | [23] |
| **Children** | 1 | 1 Yes/No | [23] |
| **Housing situation** | 1 | 1 MCQ | garden, balcony, garden and balcony, neither [23] |
| **Reason for Isolation or Quarantine** | 1 | 1 MCQ | e.g. I tested positive for the coronavirus/ I was a contact person/ I have been a contact person several times/ I was a contact person and subsequently tested positive for coronavirus/ I was a travel returnee/ Other/ I don’t know |
| **Psychological Situation** |  |  |  |
| **Psychological distress** | 5 | 6-point Likert scale | [24] |
| **Coping** | 6 | 6-point Likert scale | [30] |
| **BRS** | 6 | 6-point Likert scale | [31] |
| **Support Systems** | 2 | 1 Yes/No, 1 OQ | [32] |

Note. OQ: open question, MCQ: multiple choice question, SES: socioeconomic status
