# Supplementary material for: Psychological outcomes of depression after legally enforced quarantine during the COVID-19 pandemic: a cross-sectional study
Source: BMC Public Health. 2025 Dec 3;26:38. doi: 10.1186/s12889-025-25751-0 (PMC12766936; doi:10.1186/s12889-025-25751-0)
Supplement: Supplementary file 2 — Supplementary Material 2. [file 12889_2025_25751_MOESM2_ESM.docx]

Additional file 2: Categories of free-text answers for support systems used during quarantine

|  | **Category** | **Example** |
| --- | --- | --- |
| 1 | Therapist | “Talk with my therapist”, “Video session with my psychotherapist” |
| 2 | Semi-professional, telephonic mental health counseling | “Psychosocial service”, “Worry phone” |
| 3 | Psychological support by untrained staff | “Telephone counseling by my church”, “Online self-help program” |
| 4 | Counseling by telephone regarding symptoms | “Telephone calls with my doctor”, “116117 and a subsequent specialist consultation with a doctor” |
| 5 | Medical treatment at home | “My general practitioner came to see me after a long time” |
| 6 | Treatment in hospital/clinic | “Hospital” |
| 7 | Quarantine-specific support offered by the health department | “The emergency number of the city of Cologne”, “Citizens’ hotline of the city of Cologne”, “The coronavirus hotline of the city of Cologne” |
| 8 | Everyday support | “Delivery service” |
| 9 | Miscellaneous | “My boss often called to find out how I was doing”, “NRW [North Rhine–Westphalia] help for self-employed” |
| 10 | Friends and family | “My Children helped me” |
| 11 | None were known | “No––as I did not receive any information on who I could have contacted and how” |
| 12 | None were available | “Unfortunately, not reached, because overloaded!” |
| 13 | Not attributable |  |
