# Supplementary material for: Psychological outcomes of depression after legally enforced quarantine during the COVID-19 pandemic: a cross-sectional study
Source: BMC Public Health. 2025 Dec 3;26:38. doi: 10.1186/s12889-025-25751-0 (PMC12766936; doi:10.1186/s12889-025-25751-0)
Supplement: Supplementary file 3 — Supplementary Material 3. [file 12889_2025_25751_MOESM3_ESM.docx]

Additional file 5: Further measures wanted

| ***n* (%)** | **Total** | **Depression** | |
| --- | --- | --- | --- |
| **What further measures would you like to see or would you have liked to have seen after the quarantine period?** |  | **Yes** | **No** |
| Support from the public health department | 1,692 (36.7) | 51 (29.5) | 1,641 (37.0) |
| Testing | 854 (18.5) | 24 (13.9) | 830 (18.7) |
| Nothing | 472 (10.2) | 10 (5.8) | 462 (10.4) |
| Medical care | 471 (10.2) | 22 (12.7) | 449 (10.1) |
| Politics/society | 433 (9.4) | 18 (10.4) | 415 (9.4) |
| Length of quarantine | 123 (2.7) | 2 (1.2) | 121 (2.7) |
| Psychological support | 115 (2.5) | 17 (9.8) | 98 (2.2) |
| Financial security | 99 (2.1) | 6 (3.5) | 93 (2.1) |
| Work/education | 92 (2.0) | 5 (2.9) | 87 (2.0) |
| Securing supplies (food, etc.) | 79 (1.7) | 8 (4.6) | 71 (1.6) |
| Contact with social environment | 38 (0.8) | 1 (0.6) | 37 (0.8) |
| Miscellaneous | 30 (0.7) | 3 (1.7) | 27 (0.6) |
| Offering help/ responsibility for others | 27 (0.6) | 3 (1.7) | 24 (0.5) |
| Wishful thinking/unrealistic | 25 (0.5) | 3 (1.7) | 22 (0.5) |
| Housing situation | 17 (0.4) | 0 (0.0) | 17 (0.4) |
| Symptoms and risk factors | 11 (0.2) | 0 (0.0) | 11 (0.2) |
| Childcare | 10 (0.2) | 0 (0.0) | 10 (0.2) |
| Physical activity | 7 (0.2) | 0 (0.0) | 7 (0.2) |
| Hobbies | 4 (0.1) | 0 (0.0) | 4 (0.1) |
| Avoiding news related to COVID-19 | 1 (<0.1) | 0 (0.0) | 1 (<0.1) |

Notes. *n*_total_ = 4600; *n*_depression: yes_ = 173; *n*_depression: no_ = 4427
