# Supplementary material for: Psychological outcomes of depression after legally enforced quarantine during the COVID-19 pandemic: a cross-sectional study
Source: BMC Public Health. 2025 Dec 3;26:38. doi: 10.1186/s12889-025-25751-0 (PMC12766936; doi:10.1186/s12889-025-25751-0)
Supplement: Supplementary file 4 — Supplementary Material 4. [file 12889_2025_25751_MOESM4_ESM.docx]

Additional file 4: Categories of Support Systems Used During Quarantine

| **(n [%])** | **Total** | **Depression** | |
| --- | --- | --- | --- |
|  |  |  |  |
| **Which professional support systems did you use?** |  |  |  |
|  |  | **Yes** | **No** |
| Therapist | 62 (27.9) | 12 (42.9) | 50 (25.8) |
| Quarantine-specific support offered by the health department | 59 (26.6) | 5 (17.9) | 54 (27.8) |
| Counseling by telephone regarding symptoms | 35 (15.8) | 3 (10.7) | 32 (16.5) |
| Semi-professional, telephonic mental health counseling | 14 (6.3) | 1 (3.6) | 13 (6.7) |
| Miscellaneous | 14 (6.3) | 0 (0) | 14 (7.2) |
| Psychological support by untrained staff | 9 (4.1) | 2 (7.1) | 7 (3.6) |
| Medical treatment at home | 8 (3.6) | 2 (7.1) | 6 (3.1) |
| Everyday support | 6 (2.7) | 1 (3.6) | 5 (2.6) |
| Treatment in hospital/clinic | 4 (1.8) | 2 (7.1) | 2 (1.0) |
| Friends and family | 3 (1.4) | 0 (0) | 3 (1.5) |
| None were known | 3 (1.4) | 0 (0) | 3 (1.5) |
| None were available | 3 (1.4) | 0 (0) | 3 (1.5) |
| Not attributable | 2 (0.9) | 0 (0) | 2 (1.0) |

Notes. *n*_total_ = 222; *n*_depression: yes_ = 28; *n*_depression: no_ = 194
