# Supplementary material for: Psychological outcomes of depression after legally enforced quarantine during the COVID-19 pandemic: a cross-sectional study
Source: BMC Public Health. 2025 Dec 3;26:38. doi: 10.1186/s12889-025-25751-0 (PMC12766936; doi:10.1186/s12889-025-25751-0)
Supplement: Supplementary file 5 — Supplementary Material 5. [file 12889_2025_25751_MOESM5_ESM.docx]

*Additional file 3: Categories of free-text answers for support systems desired in the future*

| **Category** | | **Example** |
| --- | --- | --- |
| 1 | Contact with social environment | “I have more social contacts in whatever form” |
| 2 | Offering help/responsibility for others | “Study involvement, research on my person” |
| 3 | Physical activity | “I’m given tips on sporting activities” |
| 4 | Hobbies | “Visiting sauna” |
| 5 | Avoiding news related to COVID-19 | “Having watched less media” |
| 6 | Support from the public health department | “Being contacted earlier and more continuously by the health department or having the course of the disease monitored”,  “Receiving more and, above all, quicker information from the health department” |
| 7 | Medical care | “Medical aftercare”,  “Rehab in a day clinic”,  “There is a post COVID consultation appointment for all COVID positives” |
| 8 | Securing supplies (food, etc.) | “Free food delivery”,  “Food delivery service for persons in quarantine. Even if it is a paid service” |
| 9 | Financial security | “I would have had a financial buffer through state or health insurance”,  “Information on default payments for self-employed”,  “Easier financial support as a student” |
| 10 | Work/education | “2–3 working days of reintegration”,  “Better organized home office”,  “One does not have to work. Especially with a child!”,  “Being given more security and transparency in university and work” |
| 11 | Symptoms and risk factors | “That my taste and smell are coming back” |
| 12 | Housing situation | “Fiber optic cable”,  “Me living alone so as not to endanger anyone”,  “Me having a balcony to get some fresh air during the 14 days” |
| 13 | Length of quarantine | “That the quarantine period would be shorter”,  “Not to stay in quarantine for a month”,  “To shorten the quarantine period by testing negative” |
| 14 | Nothing |  |
| 15 | Politics/society | “All fellow human beings to wear nose–mouth masks without exception!”,  “There should have been better help for the events industry”,  “Clearer, faster decisions from the federal government” |
| 16 | Testing | “Free tests”,  “Another coronavirus test or antibody test would have been ordered” |
| 17 | Psychological support | “Psychological support and info videos”,  “The quarantine time is looked after psychologically”,  “One would have asked how one is doing psychologically during and after the quarantine” |
| 18 | Miscellaneous | “That these questions are asked directly after the quarantine”, “Finally, the truth comes to light and this obligation to wear masks leads to nothing” |
| 19  20 | Wishful thinking/unrealstic  Childcare | “The city of Cologne provides a Netflix subscription”, “There would not have been a quarantine”, “One would have already known more about the virus”  “That somebody takes care of my children while I am sick” |
